# Unexpected Effect of IL-1β on the Function of GABA_A_ Receptors in Pediatric Focal Cortical Dysplasia

**DOI:** 10.3390/brainsci12060807

**Published:** 2022-06-19

**Authors:** Veronica Alfano, Alessia Romagnolo, James D. Mills, Pierangelo Cifelli, Alessandro Gaeta, Alessandra Morano, Angelika Mühlebner, Eleonora Aronica, Eleonora Palma, Gabriele Ruffolo

**Affiliations:** 1 Department of Physiology and Pharmacology, Istituto Pasteur-Fondazione Cenci Bolognetti, University of Rome Sapienza, 00185 Rome, Italy; veronica.alfano@uniroma1.it (V.A.); alessandro.gaeta@uniroma1.it (A.G.); gabriele.ruffolo@uniroma1.it (G.R.); 2 IRCCS San Raffaele Roma, 00163 Rome, Italy; 3 Department of (Neuro) Pathology Amsterdam Neuroscience, Amsterdam UMC Location University of Amsterdam, Meibergdreef 9, 1105 AZ Amsterdam, The Netherlands; a.romagnolo@amsterdamumc.nl (A.R.); j.d.mills@amsterdamumc.nl (J.D.M.); a.muehlebnerfahrngruber@amsterdamumc.nl (A.M.); 4 Department of Clinical and Experimental Epilepsy, UCL Queen Square Institute of Neurology, London WC1E 6BT, UK; 5 Chalfont Centre for Epilepsy, Chalfont St Peter SL9 0RJ, UK; 6 Department of Applied Clinical and Biotechnological Sciences, University of L’Aquila, 67100 L’Aquila, Italy; pierangelo.cifelli@univaq.it; 7 Department of Human Neuroscience, University of Rome Sapienza, 00185 Rome, Italy; alessandra.morano@uniroma1.it; 8 Department of Pathology, University Medical Center Utrecht, 3584 CX Utrecht, The Netherlands; 9 Stichting Epilepsie Instellingen Nederland, 2013 SW Heemstede, The Netherlands

**Keywords:** human GABA_A_ receptor, GABA_A_ current, FCD, IL-1β

## Abstract

Focal cortical dysplasia (FCD) type II is an epileptogenic malformation of the neocortex, as well as a leading cause of drug-resistant focal epilepsy in children and young adults. The synaptic dysfunctions leading to intractable seizures in this disease appear to have a tight relationship with the immaturity of GABAergic neurotransmission. The likely outcome would include hyperpolarizing responses upon activation of GABA_A_Rs. In addition, it is well-established that neuroinflammation plays a relevant role in the pathogenesis of FCD type II. Here, we investigated whether IL-1β, a prototypical pro-inflammatory cytokine, can influence GABAergic neurotransmission in FCD brain tissues. To this purpose, we carried out electrophysiological recordings on *Xenopus* oocytes transplanted with human tissues and performed a transcriptomics analysis. We found that IL-1β decreases the GABA currents amplitude in tissue samples from adult individuals, while it potentiates GABA responses in samples from pediatric cases. Interestingly, these cases of pediatric FCD were characterized by a more depolarized E_GABA_ and an altered transcriptomics profile, that revealed an up-regulation of chloride cotransporter NKCC1 and IL-1β. Altogether, these results suggest that the neuroinflammatory processes and altered chloride homeostasis can contribute together to increase the brain excitability underlying the occurrence of seizures in these children.

## 1. Introduction

Focal cortical dysplasias (FCDs) are a group of malformations of cortical development (MCD), frequently associated with drug-resistant epilepsy, and are predominant among the pediatric population [1,2,3]. FCDs type II are the most common MCD in epilepsy surgery case series [3]. From a histopathological point of view, FCDs type II are characterized by alteration of the cortical lamination and cellular abnormalities. Furthermore, these architectural abnormalities are coupled with a “dysmature” function of the neurons found in the dysplastic areas [4]. Indeed, an anomalous synaptic transmission may be one of the key pathogenic factors leading to epilepsy in this disease. Specifically, it appears that hallmark alterations in GABAergic neurotransmission, determining its “immature” activity, have a pivotal role in this process, since GABA-mediated inhibitory neurotransmission becomes unable to regulate brain excitability and eventually even drives epileptiform activity by itself [5,6].

At present, it is a common speculation that an abnormal GABA current reversal potential (E_GABA_), mainly determined by the dysregulation of the expression of the two main cation-chloride cotransporters (NKCC1 and KCC2) [7,8], could represent one of the key pathophysiological events leading to this “depolarizing GABAergic transmission” [9,10]. Further support to this hypothesis comes from the observation that these pathophysiological mechanisms are a common feature of neurodevelopmental disorders, characterized by a high incidence of epilepsy, such as FCD [4], TSC [11], Rett syndrome [12,13] or Dravet syndrome [14].

In particular, it is widely known that pediatric FCD shows a peculiar alteration of GABAergic neurotransmission [4]. Indeed, GABA responses can behave as depolarizing, especially in the most severe cases [15], and increased GABAergic synaptic activity can be responsible of the network hyperexcitability, instead of acting as an inhibitory mechanism [16].

On the other hand, it is known that the generation and recurrence of seizures have widespread consequences on whole brain homeostasis and the onset of a “vicious cycle” of neuroinflammation is likely one of the most important factors contributing to the consolidation of the epileptogenic mechanisms [17]. In the specific case of FCD type II, recent evidence supports the hypothesis that factors contributing to the maintenance of the pro-inflammatory drive in this condition include the up-regulation of inflammatory mediators in dysmorphic cells [18], imbalance of cytokines’ regulatory networks [19] and the pro-inflammatory potential of the seizures themselves [20].

The prototypical pro-inflammatory interleukin-1β (IL-1β) has a prominent role in these inflammatory processes in both epileptic patients and animal models of epilepsy [21]. Accordingly, we have previously shown that IL-1β can decrease GABA currents amplitude in human drug-resistant temporal lobe epilepsy (TLE) by activation of IL-1β signaling [22]. Here, we investigated the effect of IL-1β on GABAergic neurotransmission in pediatric brain tissues from patients affected by FCD IIb, which is the most severe form of FCD considering the high level of neuroinflammation [18]. Indeed, even though several reports supporting the role of this cytokine in neurodevelopmental epilepsies have already been published [23,24], there is still scarce evidence regarding its potential role in the “plastic” neurotransmission that characterizes neurodevelopmental disorders.

## 2. Materials and Methods

### 2.1. Patients

The cases included in this study were obtained from the archives of the Departments of Neuropathology of the Amsterdam UMC (Amsterdam, The Netherlands) and the University Medical Center Utrecht (UMCU, Utrecht, The Netherlands). Cortical brain samples were obtained from patients undergoing surgery for drug-resistant epilepsy and diagnosed with FCD type IIb (with brain somatic mutations in the *MTOR* gene) [2]. After resection, the tissue was immediately snap-frozen in liquid nitrogen and then part of the sample was used to perform the electrophysiology experiments. All the autopsies were performed within 16 to <48 h after death, with the acquisition of appropriate written consent for brain autopsy and subsequent use for research purposes. Control autopsy cases had no known history of epilepsy, a normal cortical structure for the corresponding age and no significant brain pathology. The transcriptional profiles of post-mortem and surgical resected tissues have previously been compared, showing minimal differences if the tissue is of high quality (i.e., handled and stored as in our study) [25]. Tissue was obtained and used in accordance with the Declaration of Helsinki and the Amsterdam UMC Research Code provided by the Medical Ethics Committee. Please refer to Supplementary Table S1 for clinical details of the patients. The electrophysiology experiments were performed with three pediatric samples up to 5 years old (Table 1). However, for the bioinformatics analysis, we had a larger cohort of patients and only patients below 12 years old were considered pediatric (18 patients). Adult patients diagnosed with FCD IIb, including those 18 years old, underwent transcriptomic analysis (12 patients), whilst three of these were used for the electrophysiology experiments (Table 1). A set of electrophysiology experiments were also performed on two FCD IIa tissues (3 years old, female; 11 years old, male). Throughout the manuscript, patients are referred to with the symbol “#”, and their clinical features are summarized in Table 1.

### 2.2. Membrane Preparation

All the tissues used for electrophysiology were received in dry ice and processed immediately or stored at −80°. Preparation of human membranes and their injection in *Xenopus* laevis oocytes were performed as previously described [26]. Briefly, tissues were homogenized in a membrane buffer solution (200 mM glycine, 150 mM NaCl, 50 mM EGTA, 50 mM EDTA, and 300 mM sucrose; plus 20 μL of protease inhibitors “P2714; Sigma”; pH 9, adjusted with NaOH). Afterwards, the material was centrifuged for 15 min at 9500× *g*. Then, the supernatant was centrifuged for 2 h at 100,000× *g* with an ultra-centrifuge (Beckman-Coulter). Finally, the pellet was washed with sterile water, re-suspended in assay buffer (glycine 5 mM) and immediately used or stored at −80° until use. The use of *Xenopus* laevis frogs and the surgical procedures for oocytes extraction and use conformed to the Italian Ministry of Health guidelines and were approved by the same institution (authorization no 427/2020-PR).

### 2.3. Xenopus Oocytes Electrophysiology

All the experiments with microtransplanted oocytes were carried out 24–48 h after cytoplasmic injection [26]. GABA-evoked currents (I_GABA_) were recorded using the technique of “two-electrode voltage clamp” [27] after the oocytes were placed in a recording chamber (0.1 mL volume) and continuously perfused with oocyte Ringer solution (OR: NaCl 82.5 mM; KCl 2.5 mM; CaCl_2_ 2.5 mM; MgCl_2_ 1 mM; Hepes 5 mM, adjusted to pH 7.4 with NaOH) at room temperature (20–22 °C). GABA application was controlled through a computer-operated gravity driven multi-valve perfusion system (9–10 mL/min) (Biologique RSC-200; Claix, France). With this setup, 0.5 to 1 s are enough to completely replace the entire volume of applied solution in the recording chamber.

In all the experiments, the stability of GABA-evoked currents (I_GABA_) was evaluated on two consecutive GABA applications, separated by a 4 min washout. Only the cells that showed a <5% variation in current amplitude were used to test the effect of IL-1β. Variation in current amplitude was calculated comparing the mean current elicited by the two GABA applications before and after exposure to cytokines and/or inhibitors. GABA current reversal potential (E_GABA_) was calculated by constructing current-voltage (I-V) relationships that were then elaborated by a linear regression curve-fitting software (Sigmaplot 12, Systat software Inc., Chicago, IL, USA). In a specific set of experiments, we used IL1-Ra to block IL-1β ‘s effect. In these experiments, we pre-incubated the cells for 30 min with the blocker alone, and then proceeded to the incubation with the cytokine plus the inhibitor for two hours. For the incubation, IL-1β and IL-1Ra were diluted at the desired concentration (specified for each experiment) in Barth’s modified saline solution (88 mM NaCl; 1 mM KCl; 2.4 mM NaHCO_3_; 10 mM HEPES; 0.82 mM MgSO_4_; 0.33 mM Ca(NO_3_)_2_; 20.41 mM CaCl_2_). IL-1β was purchased from Peprotech (London, UK) and human IL-1Ra was purchased from Invitrogen (Waltham, MA, USA).

### 2.4. RNA-Seq Library Preparation and Sequencing

All library preparation and sequencing were performed at GenomeScan (Leiden, The Netherlands). The NEBNext Ultra II Directional RNA Library Prep Kit for Illumina (New England Biolabs, Ipswich, MA, USA) was used for sample processing. Sample preparation was performed according to the protocol “NEBNext Ultra II Directional RNA Library prep Kit for Illumina” (NEB #E7760S/L). The mRNA isolation was performed from the total RNA using oligo-dT magnetic beads and cDNA synthesis followed. Next, sequencing adapters were ligated to the cDNA fragments followed by PCR amplification. Clustering and DNA-sequencing were performed using the NovaSeq6000 (Illumina, Foster City, CA, USA) in accordance with manufacturers’ guidelines. All samples underwent paired-end sequencing of 150 nucleotides in length and the mean read depth per sample was 47 million reads.

### 2.5. Bioinformatics Analysis of RNA-Seq Data

The Bestus Bioinformaticus Decontamination Using Kmers (BBDuk) tool from the BBTools suite was used for adapter removal, quality trimming and removal of contaminant sequences (ribosomal/bacterial) [28]. A phred33 score of 20 was used to assess the quality of the read, any read shorter than 31 nucleotides in length was excluded from the down-stream analysis. Reads were aligned directly to the human GRCh38 reference transcriptome (Gencode version 33) [29] using Salmon v0.11.3 [30]. Transcript counts were summarized to the gene level and scaled using library size and average transcript length using the R package tximport [31]. Genes detected in less than 20% of the samples in any diagnosis and with counts less than 6 across all samples were filtered out. The gene counts were than normalized using the weighted trimmed mean of M-values (TMM) method using the R package edgeR [32]. The normalized counts were than log2 transformed using the voom function from the R package limma [33]. The subsequent differential expression was carried out using the R package limma. A linear model was fit for each gene and moderated t-statistic was calculated after applying an empirical Bayes smoothing to the standard errors. Differential expression was defined by a Benjamini–Hochberg adjusted *p*-value < 0.05. The analysis compared FCD IIb pediatric tissue samples (18 patients) and matched control cortices (6 patients).

## 3. Results

### 3.1. IL-1β Affects the GABA Current Amplitude in Oocytes Injected with FCD IIb Membranes

**Figure 1:**
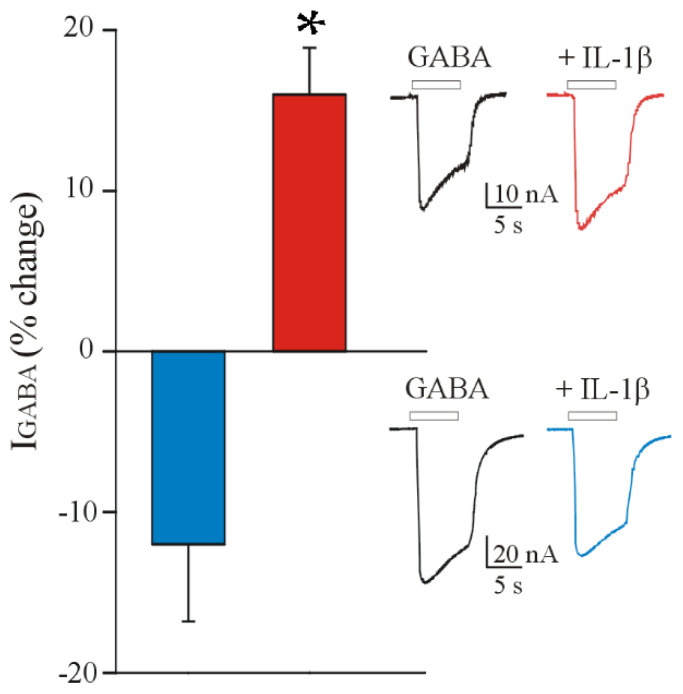
IL−1β potentiates GABA-evoked currents in pediatric FCD IIb tissue samples. The bar graphs show the average GABA current percent (%) changes ± s.e.m. before and after incubation with IL-1β (25 ng/mL; 2 h) in oocytes transplanted with adult FCD IIb tissues (blue bar; *n* = 14, # 4–6, Table 1) and pediatric FCD IIb tissues (red bar; *n* = 31, # 1–3, Table 1). *Insets* represent sample currents before (left trace) and after (right trace) incubation with IL-1β in adult (lower inset) and pediatric (upper inset) FCD IIb. White horizontal bars represent GABA application (250 μM). There is a significantly different effect of IL-1β between the two experimental groups. * *p* < 0.05 with Student *t*-test.

At first, we recorded GABA-evoked currents from oocytes microinjected with membranes from adult samples of the FCD IIb brain cortex (Table 1). We recorded responses ranging from 11.8 nA to 250.0 nA (*n* = 24; #4–6; 250 μM GABA). Subsequently, in order to test the effect of IL-1β on FCD, we measured GABA current amplitude (I_GABA_) before and after a 2 h incubation with this cytokine (25 ng/mL). We observed that this treatment induces a decrease in I_GABA_ (I_GABA_ = 98.4 ± 16.6 nA and 87.8 ± 16.8 nA, before and after IL-1β incubation, *n* = 14; *p* < 0.001, Wilcoxon signed rank test; # 3–5, Table 1). This effect was comparable to that already reported in TLE tissues [22] and behaved similarly, since IL-1β induced GABA current decrease in 70% of the cells (14/20), while the rest did not respond to the cytokine [22]. As previously shown for TLE, this I_GABA_ decrease was blocked by 30 min pre-treatment with 10 μM IL-1Ra (I_GABA_= 86.6 ± 10.6 nA and 88.7 ± 12.07 nA, before and after incubation with IL-1β + IL-1Ra, *n* = 6; *p* > 0.05, paired *t*-test; # 5, Table 1). Interestingly, when we repeated the same experiments on pediatric FCD IIb samples (range: 5.6 nA to 193.0 nA, *n* = 44; #1–3; 250 μM GABA), we did not obtain the same results, observing an opposite effect of IL-1β, which on these tissues and in the same experimental conditions reported above (2 h incubation, 25 ng/mL) induced a potentiation of I_GABA_ (I_GABA_ = 45.4 ± 10.9 nA and 52.7 ± 12.9 nA, before and after IL-1β incubation, *n* = 31; *p* < 0.001, Wilcoxon signed rank test; # 1–3, Table 1, Figure 1). Also in this case, the effect was blocked by 30 min pre-treatment with 10 μM IL-1Ra (I_GABA_ = 28.8 ± 6.2 nA and 27.6 ± 5.0 nA, before and after incubation with IL-1β + IL-1Ra, *n* = 8; *p* > 0.05, Wilcoxon signed rank test; # 1–3, Table 1). Subsequently, we repeated the IL-1β incubation experiments microinjecting the oocytes with membranes from two FCD IIa tissue samples (3-year-old female and 11-year-old male). The results showed GABA current potentiation also in these cases (I_GABA_ = 40.9 ± 8.4 nA and 47.0 ± 10.1 nA, before and after IL-1β incubation, *n* = 16; *p* < 0.01, Wilcoxon signed rank test).

### 3.2. GABA Reversal Potential in Oocytes Injected with FCD IIb Membranes

**Figure 2:**
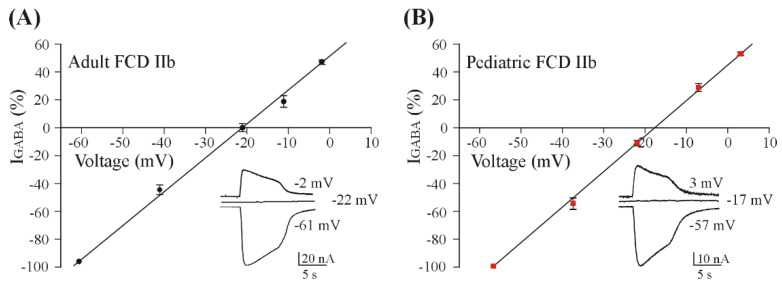
GABA reversal potential in FCD IIb tissue samples. The two panels represent the mean E_GABA_ value recorded on adult (**A**); black circles; *n* = 10, # 4–6, Table 1) and pediatric (**B**); red squares; *n* = 13, # 1–3, Table 1) FCD samples. The dots represent mean ± s.e.m. of GABA currents at correspondent holding potential value (V_H_) normalized to the maximum currents (I_max_ = 73.4 for adult and 42.4 nA for pediatric). Insets represent sample GABA−evoked currents (250 μM) at different V_H_ as shown in the respective panels. Note that the mean E_GABA_ was significantly more depolarized in pediatric FCD tissues compared to adult FCD (*p* < 0.05, Wilcoxon signed rank test).

In order to better characterize the I_GABA_ in our samples, we performed additional experiments to measure E_GABA_ in pediatric FCD IIb samples. Interestingly, we found that E_GABA_ was more depolarized in pediatric samples (−17.2 ± 1.1 mV, *n* = 13; #1–3, Table 1, Figure 2) compared to adult ones (−22.0 ± 0.73 mV, *n* = 10; *p* < 0.05, Wilcoxon signed rank test; #4–6, Table 1, Figure 2), suggesting that in pediatric patients, GABA currents have unique characteristics. Indeed, these E_GABA_ values are not comparable with those found in neurons [8] but are consistent with those previously found using the microtransplantation technique for the electrophysiological study of human brain tissue [34]. Furthermore, we repeated the same measurements in two FCD IIa pediatric samples (see methods for patients’ details) and we still found a depolarized E_GABA_, which was not statistically different compared to that recorded in pediatric FCD IIb (−16.8 ± 0.3 mV, *n* = 8, *p* > 0.05).

### 3.3. Transcriptomic Analysis

**Figure 3:**
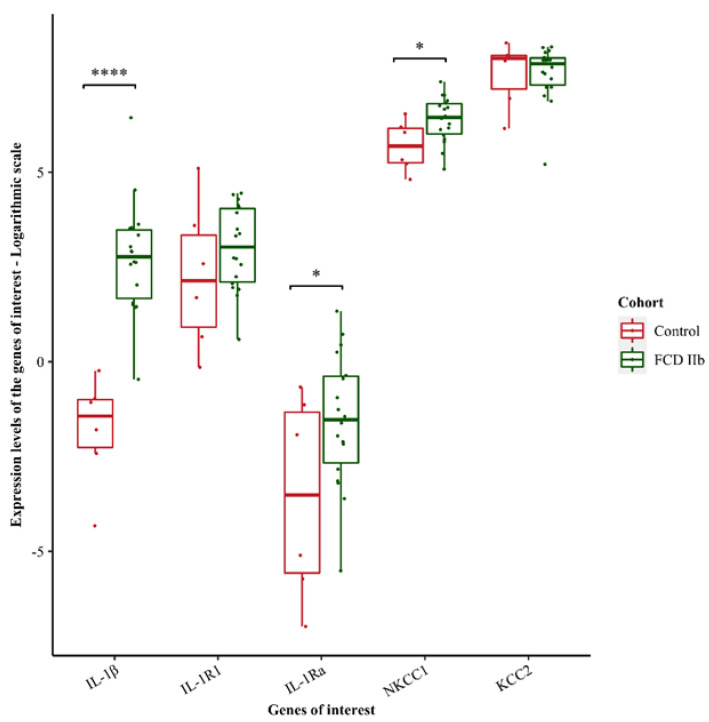
Expression levels of the genes of interest in FCD IIb pediatric tissue samples compared to age-matched controls. *IL−1β*, *IL−1Ra* and *NKCC1* are significantly upregulated in FCD IIb pediatric tissue samples compared to controls. Expression levels are described in logarithmic scale. * *p* < 0.05; **** *p*  <  0.0001. A linear model was fit for each gene and moderated t-statistic was calculated after applying an empirical Bayes smoothing to the standard errors. Those genes with a Benjamini–Hochberg adjusted *p*-value < 0.05 were considered significant. Differential expression analysis compared 18 FCD IIb patients and six age-matched control cortices.

In order to explain the aforementioned results, we performed transcriptomics analysis on a cohort of pediatric FCD IIb tissue samples. We found that the pro-inflammatory cytokine *IL-1β* and its receptor antagonist, *IL-1Ra* (IL-1 receptor antagonist) were significantly upregulated in FCD IIb pediatric tissue samples (*IL-1β* log_2_FC = 4.535; *IL-1Ra* log_2_FC of 2.042) compared to age-matched control cortices (Figure 3), suggesting *IL-1Ra* tries to counteract the overexpression of *IL-1β*. However, their common receptor, *IL-1R1* did not show a significant upregulation in these samples (log_2_FC = 0.753) (Figure 3). Interestingly, sodium-potassium-chloride cotransporter, *NKCC1*, was significantly up-regulated (log_2_FC = 0.696) (Figure 3). Altogether, these results indicate the presence of an acute neuroinflammatory process in these children and altered chloride homeostasis.

## 4. Discussion

This study focused on the analysis of the effect of IL-1β on GABAergic neurotransmission in FCD IIb samples. This cytokine can decrease GABA currents amplitude in adult FCD IIb, while it increases these responses in pediatric FCD samples. Interestingly, we found that these latter tissues were indeed characterized by a more depolarized E_GABA_ compared to adult FCD IIb, thus suggesting an alteration in chloride homeostasis [8]. Finally, the transcriptomic analysis revealed an up-regulation of the expression of *IL-1β*, *IL-1Ra* and *NKCC1* in a cohort of pediatric FCD IIb samples that fits well with the aforementioned electrophysiological results.

The technical approach we used to perform our functional experiments, membrane microtransplantation in *Xenopus* oocytes, has the potential of allowing electrophysiological recordings from rare human diseases using little amounts of brain tissue, thus making it easier to test cytokines or other mediators on these pathologies. On the other hand, with this technique, we record “whole” glial and neuronal currents without discriminating among cellular subtypes. Nonetheless, it has been demonstrated that neurotransmitter receptors transplanted in *Xenopus* oocytes from transfected cells retain their functional characteristics [35].

To our knowledge, this is the first time that IL-1β was reported to affect GABAergic function in human FCD IIb tissues, even though its ability to reduce the amplitude of GABA-evoked currents upon activation of its specific receptor was already described in human TLE [22].

Additionally, we also described the effect of IL-1β on two FCD IIa pediatric tissue samples, where we obtained the same results as the recordings in FCD IIb tissues. Indeed, FCD IIa and IIb represent two histopathological subtypes of FCD type II, and FCD IIb is further distinguished from FCD IIa by the additional presence of balloon cells [2,36]. This broadens the relevance of our findings, since we can hypothesize that the alteration in synaptic transmission by inflammatory mediators may be a general feature of FCD, at least of those forms frequently associated with drug-resistant epilepsy.

Here, we can infer that the mechanism underlying IL-1β mediated modulation of GABAergic transmission depends upon the activation of transplanted IL-1β receptors (IL-1R1) [22], since the blockade with IL-1Ra, the endogenous antagonist [37] which specifically prevents the binding of IL-β to this receptor, could prevent both GABA current decrease and increase in adult and pediatric specimens, respectively.

Alongside this mechanism, it is also possible that GABA_A_Rs modulation takes place as a consequence of the altered expression or dysregulated function of the two main cation-chloride cotransporters expressed in the brain: NKCC1, which mediates chloride influx, and KCC2, responsible for chloride extrusion [8]. Here, we described an about 5 mV E_GABA_ shift in pediatric FCD, which correlates with the reported *NKCC1* upregulation. Furthermore, this is also supported by previous findings describing a depolarizing shift of about 7–8 mV in membranes extracted from epileptic subiculum, where both an upregulation of NKCC1 and a downregulation of KCC2 were reported [34].

Interestingly, recent evidence supports the hypothesis that inflammatory stimuli, in particular IL-1β, can alter GABAergic neurotransmission also by modulating the expression and function of the aforementioned chloride transporters [38]. This is particularly relevant, since chloride homeostasis contributes to the pathogenesis of several neurodevelopmental disorders such as TSC, autism and epileptic syndromes [38]. Any kind of pathogenic factor acting on this delicate equilibrium, such as neuroinflammation, could serve as a therapeutic target for conditions that are often characterized by seizures resistant to known ASMs or medically untreatable cognitive impairment. In pediatric patients, the inflammatory processes can originate from prenatal stress, infection or traumatic injuries [38,39] that contribute to the occurrence of seizures in the first months of life. Therefore, in these patients, it could be relevant to therapeutically intervene as soon as possible to quench the neuroinflammatory processes.

Interestingly, our data indicate that *IL-1Ra* is also up-regulated, suggesting an attempt to compensate the *IL-1β* increase in our cohort of FCD IIb samples. However, the need for epilepsy surgery in these cases suggests that the endogenous anti-inflammatory cytokines failed to dampen inflammation, which can lead to brain hyperexcitability [40].

The role of GABAergic transmission in this scenario is indeed pivotal. An interesting hypothesis revolves around the ability of aberrant GABAergic transmission to promote epileptogenesis in malformed FCD cerebral cortex, especially in pediatric cases [4,6,41]. In fact, the shift in GABA reversal potential has been associated with a state of brain “dysmaturity” that is a hallmark of several neurodevelopmental diseases such as TSC [11,42], Dravet [14] and Rett syndromes [12,13].

Here, we provided novel evidence in favor of this idea, since pediatric FCD displayed aberrant, depolarized GABA responses, which were further potentiated by incubation with IL-1β.

Indeed, inflammatory mediators may be relevant for the pathogenesis of epilepsy in FCD, as confirmed by the reported up-regulation of inflammatory cytokines and their receptors and/or downstream effectors (such as IL-1β, IL-6, CCL3, CCL4, STAT3, C-JUN and CCR5) in this disease [19,43]. Hence, concerning the evident implications on the sustainment of a pro-inflammatory *milieu* and the detrimental consequences it possesses on the progression of the disease, IL-1β would also potentiate a kind of GABAergic activity that does not counteract seizures, but rather contributes to their generation and recurrence.

## 5. Conclusions

To understand the pathophysiological mechanisms underlying the dysfunction of synaptic transmission in FCD IIb is a fundamental step towards the development of new therapeutic strategies in this disease. Here, we highlighted a potential link between two relevant phenomena, such as neuroinflammation and GABAergic neurotransmission, and shed light on how the differential effect of IL-1β in pediatric versus adult tissues may depend on a disturbed chloride homeostasis in pediatric FCD IIb samples. Since endogenous anti-inflammatory response, as demonstrated by our transcriptomic analysis, may not be enough to compensate for the excessive pro-inflammatory stimuli, we can infer that an early pharmacological potentiation of anti-inflammatory mechanisms in FCD IIb may favorably affect the progression of the disease and development of epilepsy in these patients.

## Figures and Tables

**Table 1:** Clinical characteristics of the patients.

Patient	Age at the Time of the Surgery (y)	Epilepsy Onset (y)	Gender	Diagnosis	ASMs
#1	2	0	M	FCD IIb	OCZ, C, VPA,
#2	3	0	F	FCD IIb	LEV, OCZ, C, VPA, CL
#3	5	3	M	FCD IIb	C, CL
#4	18	2	M	FCD IIb	C, VPA, LMT, LCM
#5	44	10	F	FCD IIb	LEV, OCZ
#6	45	12	M	FCD IIb	LEV, OCZ

Three pediatric patients; three adult patients; Abbreviations: M = male, F = female, ASM = anti-seizure medication, C = Carbamazepine, CL = Clobazam, LCM = Lacosamide, LEV = Levetiracetam, LMT= Lamotrigine, OCZ = Oxcarbazepine, VPA = Valproic acid.

## Data Availability

Not applicable.

## Supplementary Materials

The following supporting information can be downloaded at: https://www.mdpi.com/article/10.3390/brainsci12060807/s1, Table S1: Clinical information of the pediatric study cohort used for transcriptomic analysis.
