# Supplementary material for: Unexpected Effect of IL-1β on the Function of GABAA Receptors in Pediatric Focal Cortical Dysplasia
Source: Brain Sci. 2022 Jun 19;12(6):807. doi: 10.3390/brainsci12060807 (PMC9220988; doi:10.3390/brainsci12060807)
Supplement: Supplementary file 1 [file brainsci-12-00807-s001.zip › Supplementary Table S1.pdf]

Supplementary Table S1. Clinical information of the pediatric study cohort used for transcriptomic analysis

	Diagnosis	Gender	Onset Age (year)	Age At Time of Operation (years)	Seizure Frequency (months)	Area of Resection	Mutation
1	Control	f	NA	0	NA	Cortex	NA
2	Control	m	NA	10	NA	Cortex	NA
3	Control	f	NA	0	NA	Cortex	NA
4	Control	f	NA	2	NA	Cortex	NA
5	Control	f	NA	0	NA	Cortex	NA
6	Control	m	NA	10	NA	Cortex	NA
7	FCD 2b	f	3	9	9	Frontal	MTOR
8	FCD 2b	m	3	5	152	Frontal	MTOR
9	FCD 2b	m	3	5	304	Occipital	NA
10	FCD 2b	m	1	3	152	Parietal	NA
11	FCD 2b	f	4	10	91	Temporal	TSC1
12	FCD 2b	f	0	11	17	Temporal	NA
13	FCD 2b	m	0	6	609	Frontal	NA
14	FCD 2b	m	3	8	913	Parietal	MTOR
15	FCD 2b	f	3	5	122	Temporal	NA
16	FCD 2b	f	1	9	30	Temporal	NA
17	FCD 2b	m	0	2	30	Frontal	NA
18	FCD 2b	f	1	8	30	Frontal	NA
19	FCD 2b	m	0	3	30	Frontal	NA
20	FCD 2b	f	10	10	30	Frontal	NA
21	FCD 2b	f	3	8	609	Occipital	NA
22	FCD 2b	f	0	3	517	Frontal	MTOR
23	FCD 2b	m	3	10	30	Frontal	MTOR
24	FCD 2b	m	1	2	91	Frontal	NA

Abbreviations: m=male; f=female.
